# ﻿A new species of *Chaetopelma* Ausserer, 1871 (Araneae, Theraphosidae) from Iran

**DOI:** 10.3897/zookeys.1174.109135

**Published:** 2023-08-08

**Authors:** Alireza Zamani, Rick C. West

**Affiliations:** 1 Zoological Museum, Biodiversity Unit, FI-20014 University of Turku, Turku, Finland University of Turku Turku Finland; 2 6365 Willowpark Way, Sooke, British Columbia, Canada Unaffiliated Sooke Canada

**Keywords:** Middle East, Mygalomorphae, spiders, tarantulas, taxonomy

## Abstract

New data on the tarantula genus *Chaetopelma* Ausserer, 1871 are provided. A new species, *Ch.persianum***sp. nov.**, is described based on a single female specimen collected in northwestern Iran, which represents the easternmost record of the genus within its entire known range. Additionally, the correct publication date of *Ch.olivaceum* C.L. Koch, 1842 is discussed, and the known distribution records of all *Chaetopelma* species are mapped.

## ﻿Introduction

Theraphosidae Thorell, 1869 is the largest family of mygalomorph spiders, currently encompassing over 1,000 species in 162 genera (WSC 2023). The family also includes four species known from fossils, of which *Ischnocolinopsisacutus* Wunderlich, 1988 and *Protertheraphosaspinipes* Wunderlich, 2020 represent monotypic genera from Neogene and Cretaceous Dominican amber, respectively (Dunlop et al. 2020). Commonly known as “tarantulas,” the majority of theraphosids are found in tropical, subtropical, and desert regions (Jocqué and Dippenaar-Schoeman 2006).

In the Middle East, Theraphosidae is represented by only two genera: *Chaetopelma* Ausserer, 1871 and *Ischnocolus* Ausserer, 1871. Both genera were traditionally considered closely related and part of the subfamily Ischnocolinae Simon, 1892 (Smith 1990). *Nesiergus* Simon, 1903, a small genus endemic to the Seychelles, has also been considered as sister to *Chaetopelma* (Smith 1990; Guadanucci and Gallon 2008). These proposed relationships, however, have recently been refuted. In the integrative analysis of Korba et al. (2022), *Chaetopelma* was recovered as sister to the African subfamily Eumenophorinae Pocock, 1897, and *Ischnocolus* and *Nesiergus* formed a clade with all the remaining theraphosids.

*Chaetopelma* is a relatively small genus that currently comprises six species distributed in the eastern Mediterranean and the Middle East, and one species from Cameroon (WSC 2023). A detailed taxonomic history of the genus was provided by Guadanucci and Gallon (2008). *Chaetopelma* species are fossorial tarantulas, living in burrows or within silk-lined chambers constructed beneath large stones and boulders and in the exterior dry walls of old houses and wells (Smith 1990).

In this study, we describe a new species of *Chaetopelma* collected in northwestern Iran, which represents the easternmost locality for the genus and its first recorded occurrence in Iran. Additionally, we discuss the correct publication date of *Ch.olivaceum* and provide a distribution map of all currently known species of *Chaetopelma*.

## ﻿Materials and methods

Photographs of the preserved specimen were obtained using an Olympus Camedia E‐520 camera attached to an Olympus SZX16 stereomicroscope. Digital images of different focal planes were stacked with Helicon Focus v. 8.1.1. The receptacles were photographed and illustrated after digesting tissues off in a 10% KOH aqueous solution. Body measurements exclude the chelicerae and spinnerets. Leg segments were measured on the dorsal side. Measurements of palp and legs are listed as: total length (femur, patella, tibia, metatarsus [absent on the palp], tarsus). All measurements are given in millimetres. The map was prepared using SimpleMappr (Shorthouse 2010) and modified using Adobe Photoshop. The extent of occurrence of *Ch.olivaceum* was calculated using GeoCAT (Bachman and Moat 2012). The exact geographic coordinates are omitted to prevent easy access to these places, considering the booming illegal trade with tarantulas. If precise coordinates are required for research, it is recommended to contact the institution in which the specimens are deposited and request them.

**Abbreviations: Eyes: ALE** – anterior lateral eye, **AME** – anterior median eye, **PLE** – posterior lateral eye, **PME** – posterior median eye. **Spination: Mt** – metatarsus, **pl** – prolateral, **rl** – retrolateral, **Ti** – tibia, **v** – ventral. **Spinnerets: PLS** – posterior lateral spinneret, **PMS** – posterior median spinneret.

**Depositories: NHM** – Natural History Museum, London, UK (J. Beccaloni); **ZMUT** – Zoological Museum of the University of Turku, Finland (V. Vahtera).

## ﻿Taxonomy

### ﻿Family Theraphosidae Thorell, 1869

#### ﻿Genus Chaetopelma

Taxon classificationAnimaliaAraneaeTheraphosidae

Ausserer, 1871

C26AB54F-AF5E-5361-87F6-364133BEDC47

##### Type species.

*Chaetopelmaaegyptiaca* Ausserer, 1871 by subsequent designation of Simon (1892); a junior synonym of *Ch.olivaceum* (C.L. Koch, 1842).

##### Diagnosis and description.

See Guadanucci and Gallon (2008).

##### Composition.

Eight species: *Ch.altugkadirorum* Gallon, Gabriel & Tansley, 2012 [Syria, Turkey]; *Ch.concolor* (Simon, 1873) [Turkey, Syria, Egypt]; *Ch.karlamani* Vollmer, 1997 [Cyprus]; *Ch.lymberakisi* Chatzaki & Komnenov, 2019 [Crete]; *Ch.olivaceum* (C.L. Koch, 1842) [Sudan, Middle East]; *Ch.persianum* sp. nov. [Iran]; *Ch.turkesi* Topçu & Demircan, 2014 [Turkey]; *Ch.webborum* Smith, 1990 [Cameroon].

##### Distribution.

Crete to northwestern Iran, southward to northern Sudan (Fig. 5). One species has been described from Cameroon, although it is clearly misplaced in *Chaetopelma* (see ‘Discussion’).

#### ﻿ Chaetopelma persianum sp. nov.

Taxon classificationAnimaliaAraneaeTheraphosidae

84D81DC8-3CB2-54A7-BFD9-CA19952E11F8

https://zoobank.org/2B8D36E7-CAC9-407E-B99D-1F637BB03A9C

**Figure 1.**
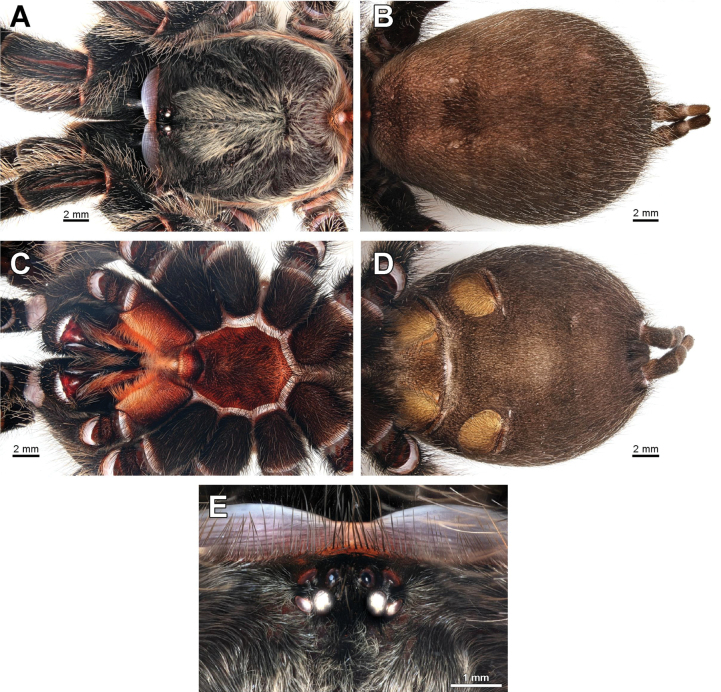
Female (holotype) of *Chaetopelmapersianum* sp. nov. **A, C** cephalothorax, dorsal and ventral views **B, D** abdomen, dorsal and ventral views **E** eye tubercle, dorsal view.

**Figure 2.**
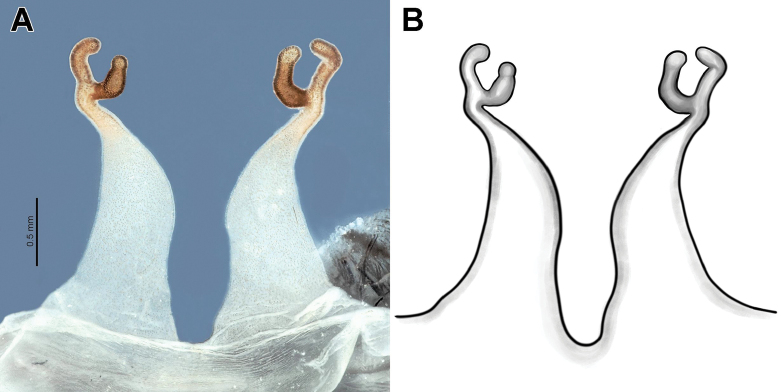
Female (holotype) of *Chaetopelmapersianum* sp. nov., receptacles **A** dorsal view **B** line drawing, dorsal view. **B** by Mahla Pourcheraghi.

**Figure 3.**
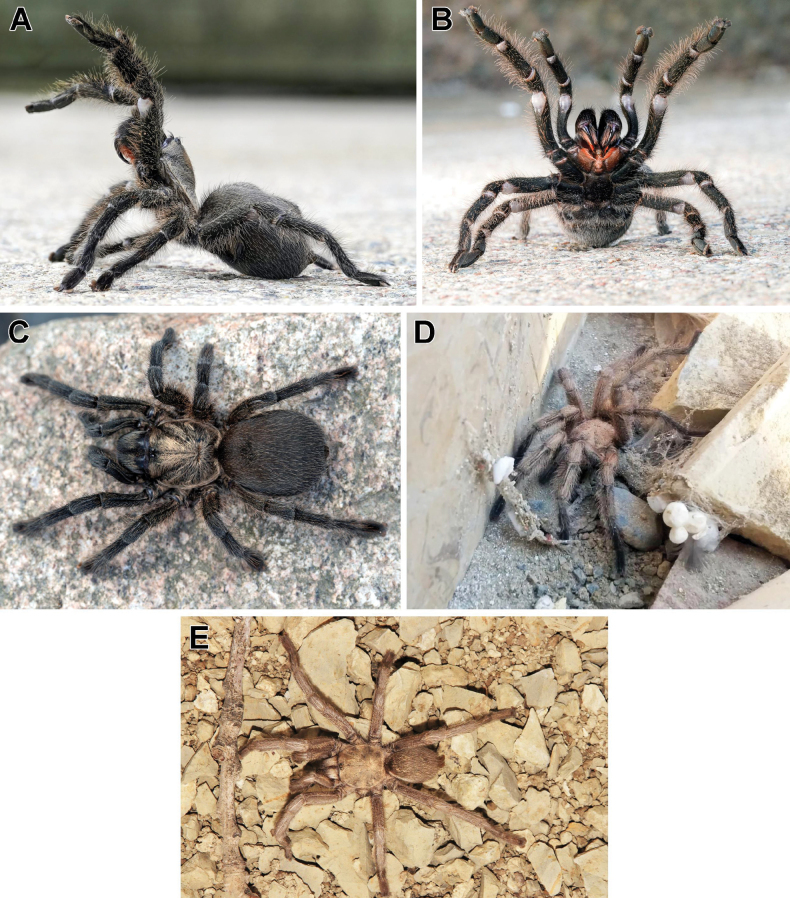
Female (holotype) of *Chaetopelmapersianum* sp. nov. (**A–C**; **A, B** in defensive posture), and unidentified males of *Chaetopelma* from Sardasht, Iran (**D**) and Sulaymaniyah, Iraq (**E**). **A–C** by Kari Kaunisto **D** by Shaton Khezrpour **E** by Christian Langner.

Figs 1A–E , 2A, B , 3A–C

##### Type material.

***Holotype*** ♀ (ZMUT), Iran: *West Azerbaijan Province*: surroundings of Mahabad, 2065 m, 26.08.2022 (A.H. Aghaei, M. Gavahyan).

##### Etymology.

The specific epithet of the new species refers to its type locality in Iran, which was historically known as Persia.

##### Common name.

We propose “Persian Gold Tarantula” (in Persian: Tārāntulā-ye Talā-ye Pārsi; تارانتولای طلای پارسی) as a common name.

##### Diagnosis.

The new species can be readily distinguished from *Ch.concolor*, *Ch.karlamani*, and *Ch.turkesi* by the bilobed apical portion of its receptacles (vs apical portion with a single lobe; cf. Fig. 2A with Chatzaki and Komnenov 2019: fig. 4C–F and Topçu and Demircan 2014: fig. 2K), in addition to being considerably larger in total body length (36.6 vs 20.5–23.5). It differs from *Ch.altugkadirorum*, *Ch.lymberakisi*, and *Ch.olivaceum* by having tubular, more elongated receptacular lobes (vs either globular, or noticeably shorter; cf. Fig. 2A with Chatzaki and Komnenov 2019: figs 4A, B, 5A–D). It can be further diagnosed from the widely distributed *Ch.olivaceum* by having shorter and less divergent receptacles (vs longer and strongly diverging posteriorly).

##### Description.

**Female.** Habitus as in Figs 1A–D, 3A–C. Total length 36.6. Carapace 13.75 long, 13.15 wide. Eye tubercle as in Fig. 1E. Eye diameters and interdistances: ALE: 0.42, AME: 0.25 (0.41), PLE: 0.41, PME: 0.38, AME–AME: 0.45 (0.35), PME–PME: 0.81. Each cheliceral furrow with 14 promarginal teeth and 12 mesobasal denticles. Labium with 73 cuspules; 1.98 long, 2.78 wide. Sternum 6.43 long, 6.30 wide. Each maxilla with ca 140 cuspules; 4.60 long, 3.05 wide; with distinct anterior lobe.

Colour in life (Fig. 3A–C): overall body and legs covered in dark brown pubescent pile setae, darker ventrally; carapace with woolly, silvery, golden pubescence; chelicerae with pale golden pile setae; labium and maxillae reddish brown; prolateral setal fringe of maxillae and along cheliceral furrows reddish; legs and abdomen covered with long scattered pale golden guard setae; book lungs light brown; spinnerets uniformly dark brown; scopulae with metallic blue-green iridescence caused by refracted light.

Colour in alcohol (Fig. 1A–C): overall as in live specimen, except for: darker and more prominent background color of body and legs; sternum, labium and maxillae more reddish; patellae with reddish brown stripes; book lungs yellowish brown.

Measurements of palp and legs: **palp**: 22.9 (8.0, 5.1, 5.5, —, 4.3), **I**: 36.35 (10.9, 7.0, 7.8, 6.4, 4.25), **II**: 33.5 (9.95, 6.05, 6.7, 5.95, 4.85), **III**: 31.0 (8.7, 5.8, 5.2, 6.85, 4.45), **IV**: 39.1 (10.75, 6.4, 7.95, 8.5, 5.5). Spination: **palp**: Ti: 1pl, 3v. **I**: Ti: 2v; Mt: 1v. **II**: Ti: 2v. **III**: Ti: 4v; Mt: 1pl, 1rl, 4v. **IV**: Ti: 4v; Mt: 1pl, 2rl, 6v. Scopulae: on metatarsi I and II very dense and covering ca 4/5, less dense on III and IV and covering distal 1/3 and 1/4, respectively; on tarsus I entire, on II–IV divided by longitudinal row of thick setae.

Spinnerets: PLS: basal article: 2.95 long, median article: 2.12 long, apical article: 2.82 long, digitiform. PMS: 1.80 long.

Endogyne as in Fig. 2A, B; receptacles paired, long, slightly diverging, and basally jointed; receptacles narrowing noticeably towards apex, each bearing two long tubular apical lobes; pore glands present all over receptacles, denser on lobes.

**Male.** Unknown.

##### Ecology.

**Figure 4.**
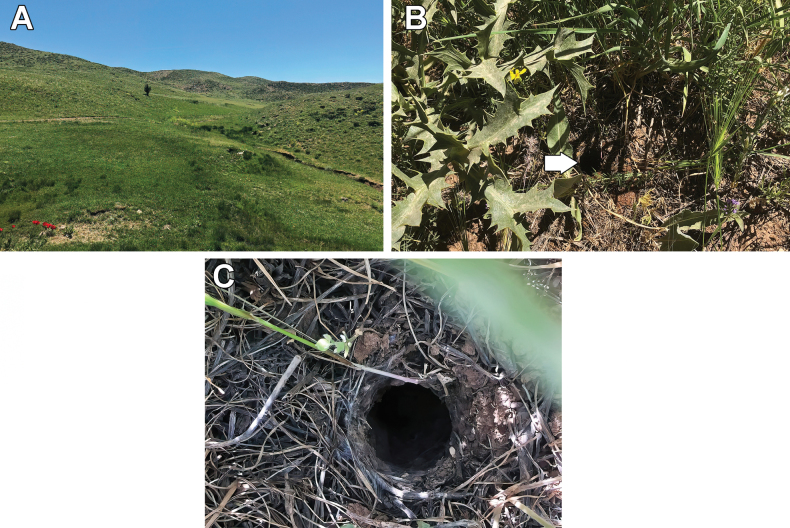
Habitat (**A**) and burrow (**B**, **C**) of *Chaetopelmapersianum* sp. nov. in West Azerbaijan Province, Iran. The arrow in **B** indicates the location of the burrow. Photos by Amir Hossein Aghaei.

An obligate burrowing species that inhabits high elevations in well-vegetated mountainous regions of the northern Zagros Mountains (Fig. 4A). The holotype was collected in a self-made ground burrow constructed on sloped rocky ground, amidst sparse low vegetation and grasses. The burrow entrance comprised a low silk collar mixed with surrounding soil and debris (Fig. 4B, C). Specifics regarding the burrow’s interior are lacking due to the use of water to extract the specimen. The rainy season spans from October to late May, with surface temperatures ranging from −8 to 14 °C. The dry season extends from June to late September, with surface temperatures ranging from 6 to 31 °C. A male was observed in the type locality in May, suggesting that the breeding season occurs towards the end of the rainy season.

##### Distribution.

**Figure 5.**
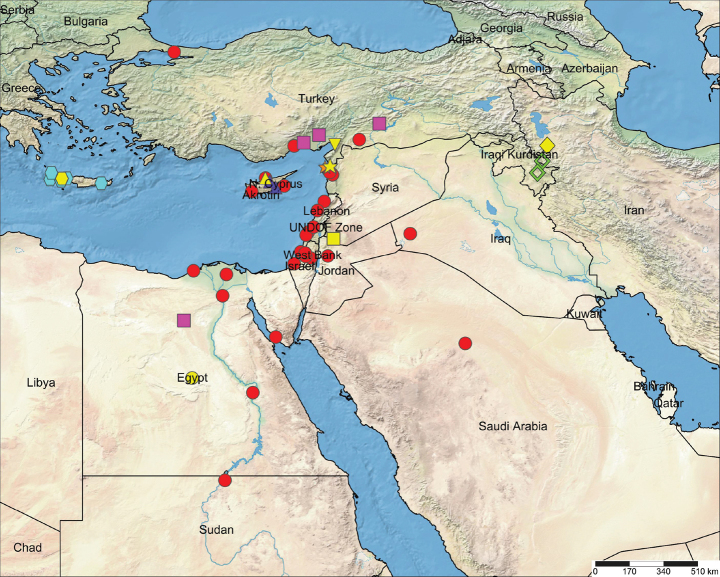
Known distribution records of *Chaetopelma* spp.: **stars**: *Ch.altugkadirorum*; **squares**: *Ch.concolor*; **triangles**: *Ch.karlamani*; **hexagons**: *Ch.lymberakisi*; **circles**: *Ch.olivaceum*; **diamond**: *Ch.persianum* sp. nov.; **inverted triangles**: *Ch.turkesi*. Yellow symbols indicate the type localities. Hollow diamonds represent localities of unidentified males that most likely belong to *Ch.persianum* sp. nov.

Currently, this species is confidently known only from the type locality, which extends the known range of the genus approximately 350 km eastwards (Fig. 5). Two males of *Chaetopelma* have been photographed in localities very close to the type locality of the new species (Fig. 5): one male in Sardasht, West Azerbaijan Province, Iran (Fig. 3D), and the other in the surroundings of Sulaymaniyah in Iraq (Fig. 3E). It is highly likely that both males belong to *Ch.persianum* sp. nov., although further study of collected material from both sexes is necessary to verify this.

## ﻿Discussion

A new species of *Chaetopelma* was described in this paper, representing the easternmost known locality of the genus and its first recorded occurrence in Iran. Currently, only two other species of Theraphosidae are known from this country, both belonging to *Ischnocolus: I*. *vanandelae* Montemor, West & Zamani, 2020, and *I.jickelii* L. Koch, 1875 (Montemor et al. 2020; Zamani et al. 2022).

Although *Chaetopelma* has undergone a comprehensive revision (Guadanucci and Gallon 2008), we believe that further investigation, particularly those employing integrative methods, would greatly benefit the taxonomy of the genus. One species in particular, *Ch.olivaceum*, exhibits one of the broadest ranges within the entire family, with an extent of occurrence of approximately 1,493,978 km^2^ (Fig. 5). As such, there is a possibility that cryptic species might exist within its expansive range. Furthermore, *Ch.olivaceum* exhibits a disjunct distribution in Turkey, being known to occur in both the southern regions of the country and as far north as Istanbul in the northwest (Fig. 5). It is plausible that integrative studies, specifically those incorporating molecular data, could reveal a distinct species status for the latter population, which is geographically isolated from the rest of the recorded occurrences. Moreover, it is expected that conducting further collection efforts in lesser-sampled or completely unexplored regions, such as Saudi Arabia, Syria, Iraq, eastern Turkey, and Iran, could lead to the discovery of additional *Chaetopelma* species or records, which would be beneficial in gaining more comprehensive insights into the taxonomy and distribution of this genus.

Almost all species of *Chaetopelma* are well illustrated, except for *Ch.webborum*, an enigmatic species known solely from the holotype female collected in Efulen (= Efoulen), Cameroon (Smith 1990). Despite Guadanucci and Gallon (2008) noting that this species does not belong to *Chaetopelma*, no further investigations have been conducted since then to clarify its taxonomic position, even though 15 years have passed since their publication. Although we were not able to study the holotype, based on the information provided in the original description, it is evident that this species is indeed misclassified. It differs from species of *Chaetopelma* in having stout and relatively short receptacles (vs long and thin), as well as pectinate tarsal claws (vs tarsal claws lacking teeth). Furthermore, its occurrence in Cameroon is significantly outside the known range of *Chaetopelma* (Fig. 5). The generic placement of this species should be revisited once the holotype specimen (NHM-03-6-30-22) becomes available for examination.

Finally, it is noteworthy that WSC (2023) has erroneously listed *Ch.olivaceum* (as well as one genus and 22 other species names) as authored by C.L. Koch in 1841, when in fact the correct date should be 1842, as noted in Simon (1892), Roewer (1942), and Bonnet (1956). In the taxonomic literature, Peters (1998) seems to be the first to incorrectly list the publication date for this species as 1841, a mistake that was later perpetuated by other researchers. This discrepancy likely stems from the WSC combining C.L. Koch’s 1842 publication with his volume from 1841, despite the clear distinction in their respective publication dates (Koch 1841, 1842).

## Supplementary Material

XML Treatment for Chaetopelma

XML Treatment for Chaetopelma persianum

## ﻿Additional information

### Author ORCIDs

Alireza Zamani https://orcid.org/0000-0002-8084-9666

Rick C. West https://orcid.org/0000-0002-1683-3395

### Data availability

All of the data that support the findings of this study are available in the main text.
